# Management of Anti-Seizure Medications during Pregnancy: Advancements in The Past Decade

**DOI:** 10.3390/pharmaceutics14122733

**Published:** 2022-12-06

**Authors:** Charul Avachat, Jessica M. Barry, Xintian Lyu, Catherine M. Sherwin, Angela K. Birnbaum

**Affiliations:** 1 Experimental and Clinical Pharmacology, College of Pharmacy, University of Minnesota, Minneapolis, MN 55414, USA; 2 Department of Pediatrics, Wright State University Boonshoft School of Medicine, Dayton Children’s Hospital, Dayton, OH 45404, USA

**Keywords:** epilepsy, anti-seizure medication, pregnancy, pharmacokinetics, post-partum

## Abstract

Management of seizures often involves continuous medication use throughout a patient’s life, including when a patient is pregnant. The physiological changes during pregnancy can lead to altered drug exposure to anti-seizure medications, increasing patient response variability. In addition, subtherapeutic anti-seizure medication concentrations in the mother may increase seizure frequency, raising the risk of miscarriage and preterm labor. On the other hand, drug exposure increases can lead to differences in neurodevelopmental outcomes in the developing fetus. Established pregnancy registries provide insight into the teratogenicity potential of anti-seizure medication use. In addition, some anti-seizure medications are associated with an increased risk of major congenital malformations, and their use has declined over the last decade. Although newer anti-seizure medications are thought to have more favorable pharmacokinetics in general, they are not without risk, as they may undergo significant pharmacokinetic changes when an individual becomes pregnant. With known changes in metabolism and kidney function during pregnancy, therapeutic monitoring of drug concentrations helps to determine if and when doses should be changed to maintain similar seizure control as observed pre-pregnancy. This review concentrates on the results from research in the past decade (2010–2022) regarding risks of major congenital malformations, changes in prescribing patterns, and pharmacokinetics of the anti-seizure medications that are prescribed to pregnant patients with epilepsy.

## 1. Introduction

The reproductive years are a critical time in the lifespan of a patient, especially for those with chronic illnesses. As the management of seizures often involves continuous medication, it is essential to understand if and how changes during distinct stages of life may affect overall drug exposure. Physiological changes during pregnancy may alter the pharmacokinetics of anti-seizure medications (ASMs), leading to fluctuations in blood drug concentrations, thus making the management of chronic epilepsy complicated. Subtherapeutic ASM concentrations in the mother may increase the seizure frequency, raising the risk of miscarriage and preterm labor. However, increased drug exposure can adversely affect the developing fetus. The crux of the management of ASM therapy in this population is balancing the number of seizures by maintaining therapeutic concentrations in the mother while minimizing fetal risk [1,2,3,4,5]. This review investigates the progress made in the past decade (2010–2022) regarding risks of major congenital malformations, changes in prescribing patterns, and pharmacokinetics of the ASMs commonly prescribed to pregnant women with epilepsy. For this review, ASMs which have been primarily used in pregnant patients have been broadly classified into older and newer ASMs based on when they were approved by the United States Food and Drug Administration (FDA) (Table 1) [6].

### 1.1. Risks of Congenital Disabilities Associated with ASM Use during Pregnancy

Approximately 1.1–1.5 million women of childbearing age in the United States (U.S.) and a further 12.5 million around the globe are affected by epilepsy [7,8]. This amounts to a lifetime prevalence of epilepsy in women of 7.62 (95% CI: (5.52–10.50)) per 1000 individuals [9]. In the U.S., 24,000 babies are born to women living with active epilepsy every year [10]. Epilepsy is also prevalent in countries outside the U.S., with Iceland and Nigeria reporting an unadjusted prevalence of approximately 3.3 (95% CI: 2.1–4.8) per 1000 pregnant women with active convulsive epilepsy [11,12]. In Italy, the prevalence of epilepsy in women of childbearing potential was shown to decline until 44 years of age, with a rise in the peri and postmenopausal age [13]. The incidence rate of epilepsy in women after pooling data from eight studies was 57.43 per 100,000 individuals (95% CI: 41.60–79.29) [9].

During pregnancy, the developing fetus of mothers with epilepsy who receive medication is exposed to ASMs in utero through the placental transport of drugs. Although taking ASMs may be necessary while a patient is pregnant, their presence may produce teratogenic effects, including major congenital malformations (MCMs). MCMs are defined as an abnormality of an essential anatomic structure present at birth that interferes significantly with function and/or requires major intervention. The MCMs frequently identified with ASM exposure include congenital heart disease, cleft lip/palate, urogenital defects, and neural tube defects [14]. In the late 20th century, pregnancy registries were established to assess the risks of in utero exposure to ASMs [15]. Infants exposed to lamotrigine and levetiracetam during pregnancy experience an MCM risk similar to that seen in infants unexposed to ASMs [14,16,17,18].

On the other hand, valproate has been shown to have the highest risk for MCM; prenatal exposure to it is associated with an increased risk of autism spectrum disorder [19,20]. Data from the North America (NAAPR) and Europe (EURAP) registries report similar risks of MCMs for most ASMs, with valproate being the highest, with 9.3% and 10.3%, respectively [16,17]. MCM risks appear to be dose-dependent for lamotrigine, valproate, phenobarbital, and carbamazepine [21]. A decline in the rate of MCMs was observed from 6% (2000–2005) to 4.4% (2010–2013) in women on ASM monotherapies observed in the EURAP registry, which has been thought to be related to changes in prescribing patterns over the past decade [18].

Long-term effects impacting the neurodevelopmental outcomes of children exposed to some ASMs have also been observed. Before the last decade, the Neurodevelopmental Effects of Antiepileptic Drugs (NEAD) study, which enrolled 305 pregnant women with epilepsy in the United States (1994–2004), indicated a negative impact on the intelligence quotient (I.Q.) of children born to mothers exposed to valproate during pregnancy. The I.Q. of children prenatally exposed to valproate at 3 and 6 years of age was lower than those exposed to carbamazepine, lamotrigine, and phenytoin [22,23,24]. These results led to changes in the recommendations of valproate use in women of childbearing age in the U.S. and Europe. The NEAD study also revealed differential risks of valproate and carbamazepine on neonatal outcomes, including birth weight and infant head size, which can help guide ASM selection in women of childbearing age [25,26].

Some newer ASMs, including oxcarbazepine, gabapentin, pregabalin, brivaracetam, lacosamide, zonisamide, and perampanel, have limited clinical data on their teratogenic potential. The EURAP registry illustrated an MCM risk of 3% associated with oxcarbazepine [17]. Pregabalin exposure during the first trimester shows an increase in the risk of MCM; however, the studies were small [27,28]. There have been conflicting reports on the teratogenic potential of pregabalin from developmental toxicity in rats at human equivalent therapeutic doses [29,30]. Preclinical studies using chick embryos and mice have illustrated that lacosamide might also have teratogenic potential, which increases in a dose-dependent fashion [31,32]. However, one study in seven pregnant women with epilepsy that collected lacosamide serum concentrations reported no MCMs in babies born to these mothers [33]. Data from the U.K. and Ireland pregnancy registers reported an MCM risk of 13% in the zonisamide monotherapy group compared to 6.9% in the polytherapy group in pregnant women with epilepsy [34]. The NAAPR registry data shows an association between zonisamide exposure and low infant birth weight compared to infants exposed to lamotrigine prenatally [35]. Brivaracetam, approved in 2016, did not produce embryo-fetal toxicity and teratogenicity in pregnant rats; however, in pregnant rabbits, embryotoxicity was observed at equivalent maternal doses [36,37]. It should be noted that enzyme systems differ across species, and the appropriateness of the animal model should be considered when interpreting teratogenicity data from preclinical animal species.

### 1.2. Prescribing Patterns of ASMs during Pregnancy

Pregnant patients with epilepsy most commonly present with focal seizures with the majority of cases being treated with ASM monotherapy [38]. Studies in the past decade have reported prescribing practices for ASMs globally. Lamotrigine is the most frequently prescribed ASM in pregnant women with epilepsy in the United Kingdom (U.K.), France, Emilia Romagna (Italy), and the Nordic countries of Denmark, Finland, Iceland, Norway, and Sweden [37,39,40]. Increases in the prescriptions of gabapentin and pregabalin were observed in the U.K. after 2012 [37,39]. In the Lombardy region of Italy, levetiracetam and lamotrigine were the most commonly prescribed ASMs beginning in 2017 [41]. The increasing trend in prescribing levetiracetam and lamotrigine is also reflected in the NAAPR [16].

Several more extensive studies in the U.S. report trends of ASM use during pregnancy. The NEAD study showed carbamazepine, valproate, phenytoin, and lamotrigine as the most prescribed monotherapies during 1999–2004. In contrast, the newer Maternal Outcomes and Neurodevelopmental Effects of Anti-Epileptic Drugs (MONEAD) study that enrolled patients between 2012–2016 revealed lamotrigine and levetiracetam as the most commonly prescribed monotherapies with co-administration of the two as the most commonly prescribed polytherapy [38]. A comparison in the prescription patterns of ASMs between 1987–1994 and 2011–2015 in India showed a significant increase in the use of levetiracetam as monotherapy and clobazam + levetiracetam + lamotrigine as polytherapy [42]. Data collected in Japan between 2005–2016 indicated a general reduction in the prevalence of prescriptions for ASMs during the first two trimesters with an increase in the third trimesters and post-partum period [43]. Anti-seizure medication use over the past decade has changed substantially in pregnant women with epilepsy, as newer drugs have been established to be safer with a less complicated pharmacokinetic profile. One of the significant changes in clinical practice has been the consideration in the prescription of valproate in women of childbearing potential reflective of its adverse effects on neurodevelopment. The prevalence of valproate prescriptions was <1 per 1000 pregnancies in 2015–2016 in the UK, France, and Emilia Romagna [37,39]. Data from the Australian pregnancy register report a declining trend in the use of valproate after 2012 [44,45,46]. On the contrary, in India (2011–2015) and Japan (2005–2016), valproate was still the most commonly prescribed ASM [42,43]. However, later reports from Japan based on data between 2016–2020 noted that levetiracetam followed by lamotrigine were the most commonly prescribed ASMs [47].

#### 1.2.1. Pharmacokinetics of AMSs during Pregnancy

During the drug development process, the characterization of the pharmacokinetics of a candidate agent can be crucial in assessing the safety and efficacy of a drug. However, many new medications are not tested in pregnant patients. In the past decade, several studies and pregnancy registries have provided data on using newer ASMs where there were previously little or no data. For example, serum concentrations of ASMs may change during pregnancy from those observed before a patient was pregnant due to various physiological changes [1]. These changes have been associated with decreased seizure control [48,49]; thus, understanding the variation in ASM pharmacokinetics during pregnancy is crucial to optimize medication use.

##### Absorption

Alteration in absorption can influence the effective dose for a patient. Absorption rate and extent can vary with formulation and between patients. Most ASMs have high bioavailability and are extensively absorbed. While some physiological changes, including decreases in gastric and intestinal motility, can occur during pregnancy [50], these are typically not enough to change a patient’s effective dose. Additional monitoring may be needed for patients who experience increased emesis during pregnancy, as the effective dose may be significantly lower due to stomach emptying. Patients who undergo emesis during pregnancy should be evaluated, and possible modifications in their treatment should be considered (i.e., the timing of doses)

##### Distribution

During pregnancy, there is a significant increase in blood volume as the fetus grows, and the pregnancy continues, increasing the volume of distribution [50] and potentially reducing ASM plasma concentrations. Additionally, albumin and alpha1-acid glycoprotein concentrations decrease throughout pregnancy due to an increase in volume, resulting in potential altered protein binding and a changed ratio of total and unbound drug concentrations. Hormones that increase during pregnancy may also compete with ASMs for protein binding. Altered protein binding is particularly significant for highly bound ASMs such as phenytoin and valproate, which have demonstrated free fraction increases that correlate with declining albumin levels [51,52,53]. However, in nonpregnant individuals dosing changes are usually unnecessary when two highly protein-bound drugs are co-administered as the effect is transient unless one of the co-medications also affects the metabolism of the other. For example, phenytoin when co-administered with a highly protein-bound P450 inhibitor such as valproate. Therefore, interpreting therapeutic drug monitoring (TDM) results can be challenging for highly protein-bound drugs, particularly those administered as polytherapy. Clinically, TDM measures total drug concentration, which could be misleading as a decrease in protein binding may manifest as a fall in total serum concentrations, whereas unbound drug concentration remains unchanged. Thus measurement of both bound and unbound drug concentrations in highly protein-bound ASMs can better characterize the pharmacokinetic changes during pregnancy [54].

##### Metabolism

Many ASMs are extensively metabolized by both cytochrome P450 enzymes (CYPs) (i.e., CYP3A4, CYP2C9, and CYP2C19) and uridine glucuronosyl transferases (UGTs). Enzyme induction occurs during pregnancy for some enzymes and is thought to be a potential culprit for the overall increase in clearance of some ASMs (Table 2). However, as an increase in clearance corresponds to a decrease in serum concentration, patients may experience a reduction in seizure control. Additionally, co-administration of other medications further complicates treatment, as some ASMs can interact with each other either by inducing or inhibiting drug metabolism.

##### Excretion

During pregnancy, kidney physiology changes dramatically. Glomerular filtration rate increases by approximately 50% [55]. Blood flow to the kidney can also increase up to 80%, altering secretion and reabsorption mechanisms [55]. Significant increases can occur in the first 20 weeks of pregnancy, resolving between 1–8 weeks post-partum [55]. Thus, ASMs cleared mainly through the renal route may have reduced serum concentrations early in pregnancy, leading to possible efficacy (Table 2).

### 1.3. Overview of Older ASMs

Older ASMs, sometimes called first-generation ASMs, can be defined as those ASMs approved before 1993. Pharmacokinetic changes during pregnancy have been characterized in many older ASMs [56,57,58,59,60]. While many of the older ASMs are no longer commonly used in women of childbearing age or during pregnancy, it is essential to understand the risks involved with their exposure. Carbamazepine clearance did not increase in 12 pregnant women; decreased concentrations were not found to be associated with reduced seizure control [61]. There is no evidence of valproate or phenobarbital clearance changes during pregnancy, although dose adjustments occur in approximately 15 and 27% of pregnancies [62].

Some of these older medications have significant risks regarding fetal exposure. Older ASMs are likely to cross the placenta in clinically meaningful amounts [63], and most have known teratogenicity. Valproate is especially problematic, and pregnancies involving valproate exposure are treated as the highest risk ASM monotherapy for major congenital malformations [64]. Due to safety concerns, valproate, phenobarbital, and phenytoin are less frequently used in pregnancy today [65] in favor of newer ASMs, including lamotrigine and lacosamide. Characterizing infant exposure to ASMs through breast milk is also essential. Older ASMs such as phenobarbital, phenytoin, carbamazepine, and valproate have a low partitioning into breast milk, but the data are reported from studies with only a few pregnancies [63,66].

### 1.4. Update on Newer ASMs

Several newer ASMs have been added to the arsenal since 1993. These medications are thought to have more favorable pharmacokinetic profiles and result in fewer potential drug interactions. Below is a summary of the newer ASMs regarding pharmacokinetics and drug exposure changes during pregnancy.

#### 1.4.1. Gabapentin

The bioavailability of gabapentin in healthy volunteers is less than 60% and decreases when higher individual doses are administered (>600 mg per dose) [67,68]. No data address the pharmacokinetic changes in gabapentin during pregnancy. Plasma protein binding is less than 3% and is expected to be similar in pregnancy. As gabapentin is not metabolized and is eliminated primarily through the renal route, changes in particular enzyme systems would not be expected to affect its clearance; however, the changes in renal function during pregnancy could lead to lower than anticipated gabapentin concentrations [69,70].

#### 1.4.2. Lacosamide

Lacosamide is 14% plasma protein bound, and 40% is excreted unchanged in the kidneys [71]. It is metabolized by several cytochrome P450 enzymes whose metabolism may change during pregnancy, including CYP2C19 and CYP3A4. Lacosamide dose-normalized concentrations decreased each trimester and may be significantly lower in the second and third trimesters compared to pre-pregnancy concentrations [33]. Recent evidence demonstrates that lacosamide dose-normalized concentration is significantly lower (up to 39.9%) during pregnancy compared to the post-partum period [72]. As seen with other ASMs, lacosamide concentrations return to pre-pregnancy concentrations after birth, although the time frame has not been characterized. Substantial distribution into breast milk has been observed, with a mean milk/serum ratio of 0.83 [71]. Serum concentrations decrease slightly during pregnancy, although these observations are based on smaller studies and indirect evidence [73]. While a patient who received 200 mg of lacosamide throughout pregnancy did not require a dose change in one case study [73], two out of seven pregnancies in seven women required dose increases due to breakthrough seizures [33].

#### 1.4.3. Lamotrigine

Lamotrigine plasma concentrations have been reported to decrease significantly (40–60%) during pregnancy, most likely due to an increase in clearance. Lamotrigine clearance changes during pregnancy may require dose modifications in patients to support seizure control [74,75,76]. Clearance change is characterized by two subpopulations, one that experiences low to no lamotrigine clearance change and includes approximately 25% of the overall population and another that experiences a high clearance change [77]. The increase in lamotrigine clearance is thought to happen early in pregnancy, as early as five weeks gestation, and can happen before a patient knows they are pregnant [78]. Plasma concentrations return to baseline post-partum within 2–4 weeks, increasing the occurrence of toxicity in mothers if treatment is not adjusted to a patient’s pre-pregnancy dose [74,77].

The timing of dose modifications during pregnancy can be challenging to determine prospectively as there is wide variability among patients. Some patients may not need a dose change during pregnancy, while others do. Further complicating treatment is that clinicians cannot determine which subpopulation a patient may fit into prospectively (low/no change in clearance versus high clearance change). The induction of UGT1A4 by estradiol is responsible for overall increases in lamotrigine clearance [79]. Changing estradiol levels during pregnancy are thought to upregulate UGT enzymes, increasing the formation of its major metabolite, lamotrigine N-2-glucuronide, through the glucuronidation pathway [79]. Indeed, an increase of 147–164% in the metabolite to parent ratio during pregnancy compared to nonpregnant baseline has been reported [80,81]. Genetic polymorphisms in UGT1A4 and UGT2B7 are also known and could play a role in the variability of lamotrigine clearance changes, specifically concerning subpopulation characterization. The exact mechanism explaining the changing clearance and the etiology of the subpopulations remains unknown. Additionally, dose-normalized concentrations of lamotrigine show significant variation depending on the sex of the fetus [82]. A ratio of pregnancy to a pre-pregnancy target concentration of <0.65 is a significant predictor of worsening seizures [83]. Although seizure worsening was not reported in the more extensive MONEAD study, it should be noted that MONEAD was an observational study with 70% of the women having increased doses during the study, thus potentially masking an increased risk of seizures with adjustments in doses to match targeted pre-conception concentrations established for individual seizure control [84].

#### 1.4.4. Levetiracetam

Levetiracetam has a high bioavailability of greater than 95%. Approximately 24% of the levetiracetam dose is metabolized by enzymatic hydrolysis, forming an inactive metabolite. It is minimally protein bound and is eliminated primarily through the renal route [85,86]. Levetiracetam clearance increases during pregnancy; however, results are inconsistent regarding which trimester clearance changes occur, most likely due to the small sample sizes in studies. Levetiracetam clearance is reported to be higher during pregnancy than post-partum or pre-pregnancy baseline concentrations, with changes experienced during the first trimester as observed by lower levetiracetam dose-normalized concentrations in a more extensive study of 151 pregnant women with epilepsy [49,72,87,88,89]. The early increase in clearance is believed to be due to an increase in renal blood flow and glomerular filtration rate reported during pregnancy [55,90,91]. A summary of apparent clearance, dose changes, and information on seizure frequency during pregnancy for women taking lamotrigine and levetiracetam are presented in Table 3.

#### 1.4.5. Topiramate

Topiramate is readily absorbed and highly bioavailable but is not significantly metabolized. It is mainly excreted unchanged in the urine in nonpregnant individuals [91]. Although there is wide variability in serum concentrations, they are similar or decreased after the first trimester, thought to be due to increased glomerular filtration [56]. Topiramate dose to concentration ratios have increased throughout pregnancy, with an average increase of 71.8% during the third trimester compared to the nonpregnant baseline [92]. In a study of 15 women, seizure frequency increased in 47% of pregnancies. Consequently, dose increases and the addition of ASMs were seen in the second and third trimesters [56]. The mechanism of these changes is not fully characterized, as there are conflicting findings regarding topiramate metabolism during pregnancy.

#### 1.4.6. Oxcarbazepine

Oxcarbazepine is rapidly metabolized to its active 10-monohydroxy metabolite (MHD), which is thought to be responsible for most anti-seizure effects [92]. Parent and metabolite have half-lives of 2 and 9 h, respectively. MHD binds moderately to albumin (40% bound). Both total and unbound dose-corrected MHD concentrations are decreased (up to 32.6% and 30.6%, respectively) during pregnancy and return to baseline post-partum [72]. There is significant variability in the magnitude of changes, with some studies reporting changes of up to 64% by the third trimester, while other studies report no significant differences across trimesters [93,94]. Some patients experience deterioration of seizure control following decreased concentrations [93,94,95]. Doses were changed on average by 1.5 fold in 14 pregnancies compared to the pre-conception dose in almost half of the pregnancies (~43%) [96]. Despite increasing doses, a 12–20% decrease in plasma concentrations can occur over each trimester [49]. Additionally, while loss of seizure control has been frequently observed, study populations were not large enough to quantify the association between decreasing concentrations and increased seizure frequency [49,96].

#### 1.4.7. Pregabalin

The oral bioavailability of pregabalin is greater than or equal to 90% and is dose-independent. Food negatively impacts the absorption rate, resulting in the maximum concentration decreasing by approximately 25% to 30%, and the time to maximum concentration increased by approximately one hour. Pregabalin does not bind to plasma proteins in nonpregnant patients. Approximately 90% of the administered dose is eliminated renally [70,97]. Thus, an increase in renal clearance during pregnancy may result in lower pregabalin concentrations. Comparable to gabapentin, no data address the pharmacokinetic changes in pregabalin during pregnancy.

#### 1.4.8. Zonisamide

Zonisamide is primarily renally eliminated, with 62% excreted unchanged in the urine and approximately 40% bound to plasma proteins. Although there is limited data on pharmacokinetic changes with zonisamide during pregnancy, there is evidence that serum concentrations decrease by over 40% during pregnancy, which may lead to increased seizure frequency [49,97,98].

### 1.5. Pharmacokinetics of ASMs Released in the Last Decade

Five ASMs were approved in the past 10 years: ezogabine, eslicarbazepine, perampanel, brivaracetam, and cannabidiol. There are no published studies in pregnant patients investigating the pharmacokinetics of ezogabine, eslicarbazepine and cannabidiol. One study of eslicarbazepine demonstrated inconclusive evidence regarding teratogenicity [99]. A case report of three pregnant patients taking brivaracetam shows three minor congenital malformations in two infants but no MCMs. In a published case series, one patient prescribed brivaracetam monotherapy demonstrated stable brivaracetam concentrations throughout pregnancy [71]. A second patient on lacosamide, perampanel, and brivaracetam polytherapy showed stable lacosamide and brivaracetam concentrations. However, the concentration of perampanel steadily increased during pregnancy and resolved post-partum. Perampanel concentration has also been explored in silico. Simulated concentrations from a physiologically based pharmacokinetic model of perampanel during pregnancy [100] indicate a decrease in drug exposure of three to four-fold throughout pregnancy. Data available through registry records indicate that out of the 43 full-term pregnancies exposed to perampanel. Five infants experienced adverse events [100]. Even though no MCMs were noted, the authors interpret the safety of perampanel with caution due to the low number of pregnancies. For cannabidiol, there are no published pharmacokinetic data during pregnancy in patients with epilepsy and minimal data in healthy populations. Absorption is widely variable, particularly with the wide range of formulations. Cannabidiol is metabolized by the cytochrome P450 enzyme system, particularly CYP3A4, which demonstrates altered activity during pregnancy [101,102]. Studies of larger populations are needed to elucidate if there are any pharmacokinetic changes in the more recently approved medications during pregnancy.

### 1.6. Management of ASMs during Pregnancy

The mechanism of change in overall exposure to ASMs during pregnancy vary among drugs. The management of ASMs often requires trial and error to achieve a stable optimal dose for a particular patient, which involves creating a balance between seizure frequency and adverse events of ASM therapy. One of the most extensive studies to date to include ASM concentrations, the MONEAD study, shows no significant difference in the seizure frequency for any ASM between pregnant women with epilepsy and nonpregnant women in the nonpregnant epilepsy control group. However, 74% of pregnant women with epilepsy had at least one dose modification during pregnancy compared to 31% of the nonpregnant women in the epilepsy control group [84].

Regular TDM is often employed to minimize fluctuations resulting from significant changes in drug clearance, such as those experienced when a patient becomes pregnant. The Epilepsy Birth Control Registry, which enrolled 1144 women of childbearing age with epilepsy, reported that approximately 79% of this population had at least one unintended pregnancy in the past decade [103]. Failure of contraceptive pills is one of the four leading causes of unplanned pregnancies in patients of childbearing potential [104,105]. Clinical management of epilepsy during pregnancy often requires more frequent dose adjustments as the person progresses through each trimester due to the physiological changes occurring throughout pregnancy. Evidence exists for significant increases in the clearance of some ASMs, resulting in decreased blood concentrations and potentially a subsequent increase in the seizure frequency.

As some pharmacokinetic changes happen early in pregnancy before a patient knows they are pregnant, establishing an individual patient’s optimal ASM and dose before pregnancy is essential. Avoidance of certain ASMs in patients of childbearing potential may be warranted as valproate is not recommended, although it is essential to point out that valproate may be appropriate for some patients as it may be needed for the management of seizures [106,107,108]. Therefore, the choice of ASM in patients with childbearing potential is vital, as patients do not always know when they will become pregnant.

Currently, there are no clinical guidelines for adjusting doses when a patient becomes pregnant. While some literature exists suggesting a possible algorithm for dosing changes, there is no data on the robustness of these approaches. The personalized medicine movement, along with other accelerating fields, has driven the adoption of the importance of therapeutic drug monitoring, particularly during pregnancy.

**Table 2:** Metabolism and excretion changes influencing ASM concentrations.

	Parameter	Change in Activity	Theoretical—ASMs Affected	ASMs with Clinical Evidence in Pregnancy
Metabolism [109,110]	CYP1A2	Decreased in all trimesters [111,112]	Perampanel [71]	---
	CYP2D6	Increased in all trimesters [111]	---	---
	CYP2C9	Increased in trimesters 2 and 3 [113]	phenobarbital, primidone, valproate [114]	
	CYP2C19	Decreased [115,116]	phenytoin [117,118], lacosamide	Phenytoin [119]
	CYP3A4	Increased in the third trimester [111,112]	Carbamazepine [120], zonisamide [121], lacosamide, perampanel [122]	Carbamazepine [120]
	UGT1A4	Increased in trimesters 2 and 3	lamotrigine [79], eslicarbazepine [123]	Lamotrigine [74,75,76]
	UGT1A1/9	---	Ezogabine [124]	---
Excretion	Renal blood flow	Increased	topiramate, levetiracetam, pregabalin	Topiramate [56,92]

**Table 3:** Summary of clearance changes, dose, and seizure frequency of levetiracetam and lamotrigine in women with epilepsy.

Study	M/P	N	Baseline (PC/PP)	Baseline Apparent CL	Change from Baseline/CL during Pregnancy			Dose at Baseline (mg)	Dose Increase from Baseline/Dose during Pregnancy				Seizure Frequency Change from N.P. Baseline (% of the Population)			Other Findings
					TM1	TM2	TM3		TM1	TM2	TM3	PP	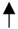	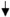	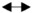	
LEVETIRACETAM																
Reisinger et al. [49]	M	15	P.C.	1.09 ^a^	98% 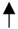	207% 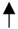	97% 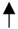	2395.8 ^a^	3%	29%	41%	-	47%	27%	27%	Seizure worsens when ASM blood concentrations decrease > 35% P.C. concentrations
Voinescu et al. [87]	M/P	16	PC/PP ^c^	-	1.71-fold	1.42-fold	1.37-fold	-	-	-	-	-	-	-	-	-
Tomson et al. [88]	M/P	12	PP ^c^	124.7 ± 57.9 L/day ^a^			427.3 ± 211.3 L/day ^a^	2000 ^a^	-	-	No increase observed	-	-	29% ^d^	71% ^d^	25% of subjects had an increase in the P.P. dose compared to TM3.
Berlin et al. [125]	-	59	PC	166.48 L/day ^a^	237.56 L/day ^a^	236.02 L/day ^a^	242.56 L/day ^a^	2051.3 ^a^	2149 mg	2538.6 mg	2770.3 mg	2703.1 mg ^e^	-	-	-	P.P. apparent CL was 107.8 L/day and was significantly different from the pre-pregnancy value. However, no significant difference in mean apparent CL between trimesters was observed.
Yin et al. [126]	M/P	26 (M+P)	PC/PP	90 L/day ^a^	200 L/day ^m^	250 L/day ^a^	240 L/day ^m^	1000 mg ^I,m^	1000 mg ^l,m^	1500 mg ^l,m^	1750 mg ^l,m^	-	53.3% (in monotherapy group)	-	-	Significant differences in the RTC values between patients with and without seizure worsening was not observed
LAMOTRIGINE																
Reisinger et al. [49]	M	69	PC	0.87 ^a^	89%	191%	140%	406.5 ^a^	5%	39%	63.5%	-	38%	12%	51%	Maximum apparent CL change from 1st to 2nd pregnancy on 15 subsets of women on LAMOTRIGINE monotherapy ranged from 69% decrease to a 76% increase.
Reimers et al. [80]	-	19	PP	1.7 ± 0.7 L/h ^b^	-	-	3.7 ± 1.5 L/h ^b^	255 ± 144 (at conception) ^b^	-	-	34%	-	-	-	-	118% increase in apparent clearance in TM3 as compared to P.P. Increase in LAMOTRIGINE-GLUC/LAMOTRIGINE serum concentration ratio during pregnancy was positively correlated with estradiol concentration and was 164% higher than baseline in the 8th month of gestation.
Tran et al. [74]	M/P	12 ^f^	PC	1.2 L/(kg·day) ^a^	1.97 L/(kg·day) ^a^	2.31 L/(kg·day) ^a^	2.26 L/(kg·day) ^a^	11 out of 12 pregnancies needed a dose increase					-	-	-	A significant difference in the apparent LAMOTRIGINE CL when TM2 and TM3 values were compared to P.P. and P.C. values.
Pennell et al. [83]	M/P ^g^	53	PC/PP ^h^	Total and free LAMOTRIGINE CL at TM1,2,3 significantly different than N.P. baseline			94% ^i^/ 89% ^j^	-					39%	33%	28%	The ratio to target concentration <0.65 is a significant predictor of worsening seizures. Total and free LAMOTRIGINE CL at TM 2 and 3 significantly different than TM1
de Haan et al. [127]	M	12	PC	^-^	-	-	-	^-^	-	-	-	-	75%	-	-	A decline in the dose-normalized LAMOTRIGINE concentration ratio (40% of baseline) was observed
Ding et al. [128]	M	12	PC	40.5 ± 12.8 (mg/[mg/L]) ^b^	82.5% 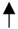	203.2% 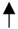	197% 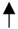	92.8 mg	-	-	-	-	The maximum population (57.1%) experienced an increase in seizure frequency during 5th month of pregnancy			Ratio to target concentration <0.64 is a significant predictor of worsening seizures
Ohman et al. [81]	M/P	15 ^k^	PP ^c^	66.5 ± 17.9 L/day ^b^	~120% 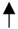	~230% 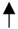	250% 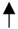	304.4 mg ^a^	283.3 mg ^a^	327.1 mg ^a^	355.8 mg ^a^	Baseline	9/17 women experienced seizures during pregnancy. No comparison with baseline reported			lamotrigine-2-N-glucuronide/lamotrigine plasma concentration ratio was 0.349 ± 0.141 (±SD) at NP baseline which increased by 147% in late pregnancy.
Fotopoulou et al. [129]	M	9	PC/PP	39 L/kg ^l^	197% 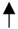	236% 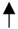	248% 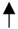	The average LAMOTRIGINE dose increase was 250% during pregnancy					-	-	-	A 264% increase in median LAMOTRIGINE apparent CL was observed at delivery.
Yin et al. [126]	M/P	25 (M+P)	PC/PP	52.2 L/day ^a^	150 L/day ^m^	178.6 L/day ^a^	175 L/day ^m^	100 mg ^I,m^	137.5 mg ^m^	168.8 mg ^m^	275 mg ^m^	-	47.6% (in the monotherapy group)	-	-	Significant differences in the RTC values between patients with and without seizure worsening was not observed

N = Number of individuals, CL = Clearance, TM1 = Trimester 1, TM2 = Trimester 2, TM3 = Trimester 3, PP = Postpartum, NP = Non -pregnant, M = Monotherapy; P = Polytherapy, CL = Clearance, PC = Preconception. a = Mean, b = Mean +/− SD, c = >4 weeks PP, d = Data from 7 out of 12 patients, e = PP was collected 2–12 weeks post-delivery, f = From 11 women, g = Analysis of seizure frequency was done with patients on monotherapy with epilepsy diagnosis, h = > 6 weeks PP, i = Mean total LAMOTRIGINE CL, j = Mean free LAMOTRIGINE CL, k = 15 women contributed to 17 pregnancies; only 12 pregnancies used for CL analysis, l = Median, m = based on approximation from information in reference tables/figures, - = no mention of data in paper. 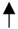 = Increase, 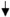 = Decrease, 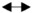 = No change.

## 2. Conclusions

The past decade has seen the adoption of treatment for seizure control with lower-risk ASMs during pregnancy, in turn supporting maternal and fetal health. However, many pregnancies are unplanned, which makes the choice of ASM initiated in patients of childbearing age crucial. Regular TDM and dosage modification can aid in the adequate management of seizures and minimize ASM exposure to the fetus, thus enhancing the safety of the ASM in the mother and child. Further research on a greater number of pregnant individuals taking newer ASMs is required to understand how pharmacokinetic changes during pregnancy might affect the developing fetus or seizure control in patients. Therefore, long-term studies that report on child outcomes are essential to determine the overall repercussions of treatment with some ASMs over others.

## Figures and Tables

**Table 1:** Anti-seizure medications used in pregnant patients.

Older (before 1993)	Newer (after 1993)
phenobarbital	gabapentin
phenytoin	lamotrigine
carbamazepine	topiramate
primidone	oxcarbazepine
valproate	levetiracetam
	zonisamide
	pregabalin
	lacosamide
	perampanel
	brivaracetam

## Data Availability

Not applicable.
